# Multiple family therapy for anorexia nervosa at the Eating Disorder Service, the Children's Hospital at Westmead

**DOI:** 10.1186/2050-2974-3-S1-O11

**Published:** 2015-11-23

**Authors:** Andrew Wallis, Julian Baudinet, Lisa Dawson, Elaine Tay, Dale Greenwood, Caitlin McMaster, Jane Miskovic-Wheatley

**Affiliations:** 1 Eating Disorder Service, The Children's Hospital at Westmead, Australia

## 

The Eating Disorder Service at The Children's Hospital at Westmead (CHW) is a tertiary service that offers a range of family focused treatment options for young people with an eating disorder. Multiple Family Therapy (MFT) is the newest treatment option provided by the service. MFT is now a key intervention offered by a number of services overseas, most notably at the Maudsley Hospital, London, where the model was developed. Despite it's use for more than a decade overseas, we are the first service in Australia to systemically integrate MFT as an additional treatment option within the standard suite of interventions offered.

MFT theoretically builds upon the core constructs of family based treatment for anorexia nervosa, whilst adding the unique experience of solidarity for young people and their families. The content of MFT is experiential, involving activities and specific debriefing techniques to help families develop ways to work together against anorexia, increase attunement to their child's needs and feel more agency around the process of recovery.

The MFT program at CHW provides the opportunity for up to eight families to work together for a 4-day workshop. Follow-up care is provided by outpatient family therapy or integration into the Intensive Family and Adolescent Eating Disorders Day Program.

MFT targets families not progressing in outpatient care or who present with some other complexity with the view that engagement in treatment may be enhanced through the group experience of MFT and the opportunity to receive treatment input from multiple sources.

The presentation will describe MFT constructs, the programs implementation at CHW, show material from the therapeutic activities completed and present preliminary data and family experiences from the first five groups.

